# Learning From the Past to Improve the Future: Value-Added Services as a Driver for Mass Adoption of Contact Tracing Apps

**DOI:** 10.1007/s12599-022-00742-2

**Published:** 2022-02-14

**Authors:** Dana Naous, Manus Bonner, Mathias Humbert, Christine Legner

**Affiliations:** Faculty of Business and Economics (HEC), University of Lausanne, 1015 Lausanne, Switzerland

**Keywords:** Contact tracing, Mobile app design, Conjoint analysis, Privacy design, COVID-19

## Abstract

Contact tracing apps were considered among the first tools to control the spread of COVID-19 and ease lockdown measures. While these apps can be very effective at stopping transmission and saving lives, the level of adoption remains significantly below the expected critical mass. The public debate as well as academic research about contact tracing apps emphasizes general concerns about privacy (and the associated risks) but often disregards the value-added services, as well as benefits, that can result from a larger user base. To address this gap, the study analyzes goal-congruent features as drivers for user adoption. It uses market research techniques – specifically, conjoint analysis – to study individual and group preferences and gain insights into the prescriptive design. While the results confirm the privacy-preserving design of most European contact tracing apps, they emphasize the role of value-added services in addressing heterogeneous user segments to drive user adoption. The findings thereby are of relevance for designing effective contact tracing apps, but also inform the user-oriented design of apps for health and crisis management that rely on sharing sensitive information.

## Introduction

The COVID-19 pandemic has created a state of emergency in countries around the world. In the early phase of the pandemic, governments considered contact tracing apps as one of the most promising tools to fight the virus and prevent lockdown measures. Corona-Warn-App in Germany, SwissCovid in Switzerland, and TousAntiCovid in France are just a few of the national apps that were developed and launched in 2020. Despite the high expectations, the adoption rates in most countries remained far below the threshold of 60% which corresponds to the desired percentage of a country’s population using contact tracing apps for them to be effective (University of Oxford 2020). In Europe, France with 47% (Rodgers 2021), UK with 40% (NHS 2021), and Germany with 39% (RKI 2021) boast the “best in class” adoption rates in 2021, although they still fail to meet expectations in terms of critical mass.

In many countries in the West, the introduction of contact tracing apps has been accompanied by controversial debates about their privacy implications and the risk of surveillance and revealed ethical or moral dilemmas (Rowe 2020). Accordingly, earlier research on contact tracing apps has mostly focused on the technology design for privacy-preserving apps (Ahmed et al. 2020; Cho et al. 2020; Yasaka et al. 2020). Because of the slow adoption rates, von Wyl et al. (2020) called for more research on the acceptability of contact tracing apps to provide an understanding of the rationale behind their use. Some of the first empirical studies that responded to this call are Trang et al. (2020), who analyze the impact of various app specifications (i.e., benefit appeal, privacy design, and convenience design) on app acceptance, as well as Meier et al. (2021) and Welrave et al. (2020), who examine users’ intentions to use contact tracing apps in light of privacy concerns. Other studies have analyzed how more users can adopt contact tracing apps. For example, Buder et al. (2020) apply choice experiments to assess users’ preferences for additional benefits, such as priority testing and food delivery, for increased uptake. Similarly, Jonker et al. (2020) assess the role of financial incentives on users’ adoption of contact tracing apps. Although these studies highlight that offering extended services can lead to a larger user base, their core focus remains on privacy design, which is reflected in the current app designs worldwide. Increasing adoption of contact tracing apps has proved to be a challenge, and involving users in the discussion on app characteristics and aspects related to the data processing is critical to ensuring mass acceptance (Redmiles 2020). This was also highlighted by Gupta and De Gasperis (2020), who suggest participatory design with users to help ensure that contact tracing apps meet their needs and are usable. While Trang et al. (2020) suggest there should be a one-size-fits-all app, their results also show that users’ preferences are far from uniform. In IS research, the privacy calculus explains the intention to use as the result of privacy trade-offs between expected benefits and perceived privacy risks (Dinev and Hart 2006). Thus, we anticipate an opportunity to address the varying preferences of different segments of the population with more targeted features that would provide both public and individual benefits. Wortmann et al. (2019) suggest that goal-congruent features, which are additional features on top of the core system functionality, can result in higher system use, regardless of its core features. For contact tracing apps, this implies that more attention is needed to provide services that offer benefits to users and can result in a larger user base.

This motivates our research goal to explore the role of goal-congruent features in improving the design of contact tracing apps and as an adoption driver. More specifically, we study the following question:

What are users' preferences for contact tracing app features, and what is the impact of value-added services on users’ adoption of these apps?

Based on a conjoint analysis (CA) study in Germany, we provide empirical insights into users’ preferences for core and privacy-preserving features, as well as value-added services of contact tracing apps. As an established market research technique, CA is a “practical set of methods for predicting consumer preferences for multi-attribute options in a wide variety of product and service contexts” (Green and Srinivasan 1978, p. 103). Occasionally, it has been used to study privacy trade-offs in the design of personal ICTs (Mihale-Wilson et al. 2017; Naous and Legner 2019). Our results confirm the dominant privacy-preserving design of most national contact tracing apps in Europe but also contribute to a more nuanced understanding of individual and group preferences. Following market simulations, we find that goal-congruent features – specifically, value-added services with a clear benefit structure – play an important role in driving user adoption.

From our study, we gain insights for a prescriptive design that allow the formation of app features that fit users’ expectations, with implications for service providers to adjust their offerings to different user segments. Our contributions are two-fold: first, our findings emphasize the role of goal-congruent features in addressing heterogeneous user segments with different benefit-risk tradeoffs and thereby fostering mass adoption. Second, methodologically we demonstrate that conjoint analysis – specifically, market simulation techniques – allow us to explore user preferences as a complementary method for participatory app design. Thus, our study contributes to the design efforts of contact tracing apps in particular, but more generally inform the user-oriented design of apps for digital health and crisis management that rely on sharing sensitive information.

The rest of the paper is structured as follows: First, we provide background on contact tracing in the context of COVID-19. Then, we introduce the applied research methodology, followed by a detailed description of the design of the CA. Next, we present the empirical results. Finally, we discuss our findings and conclude with implications for research and practice.

## Background

### Contact Tracing and Disease Control

Contact tracing is a key control measure in the battle against infectious diseases and, when systematically applied, can break the chain of transmission (Feretti et al. 2020). The World Health Organization (WHO) defines contact tracing as “the process of identifying, assessing, and managing people who have been exposed to a disease to prevent onward transmission” (WHO 2018, p. 2). Contact tracing is an extreme form of locally targeted control and can be highly effective when dealing with a low number of cases(Eames and Keeling 2003). It has traditionally been carried out by health authorities using expert-led interview-based techniques, which requires availability of human resources and subject to recall bias where not all contacts might be identified (O’Connell et al. 2021). In the case of COVID-19, contact tracing requires identifying people who may have been exposed to the virus and following up with them every day for at least 14 days from the last exposure (Legendre et al. 2020). Because symptom onset may only occur days after infection, it is difficult for traditional approaches to map the network of potential exposure traces and, thus, control the transmission rate of the virus. Therefore, advanced techniques are required for effective contact tracing in the COVID-19 context.

### Contact Tracing Apps for COVID-19

Amid the COVID-19 pandemic, governments and health authorities around the world developed mobile applications that enable digital contact tracing as a fast and reliable way to support the public health authorities’ traditional approaches. These contact tracing apps continuously track users’ proximity and, in the event of possible COVID-19 exposure, notify them that they should self-isolate (Feretti et al. 2020; Legendre et al. 2020). Simulations confirm that if approximately 60% of the population uses the national contact tracing app, it is possible to stop the epidemic and keep countries out of lockdown (University of Oxford 2020). In reality, however, adoption rates remain significantly below this threshold in almost all countries (see Table 1).

**Table 1:** Overview of contact tracing apps (as of September 2021)

App (by country)	Launch date	Users* (Sept. 2020)	Users* (Sept. 2021)	Approach	Technology	User identification	Value-added services
TraceTogether (Singapore)	20 March 2020	+ 2 M (42%)	+ 4.8 M (90%)	Centralized	Based on legacy BLE	Phone number required	SafeEntry integration Physical tokens
Hamagen (Israel)	22 March 2020	+ 1.5 M (17%)	+ 2.5 M (27%)	De-centralized	Cross-referencing of GPS data	No information required	Safe places
StoppCorona (Austria)	25 March 2020	+ 0.7 M (8%)	+ 1.3 M (14%)	De-centralized	Based on legacy BLE	Phone number required	Symptoms check
COVIDSafe (Australia)	26 April 2020	+ 7 M (28%)	+ 7 M (28%)	Centralized	Based on legacy BLE	Personal information required	Information center/ Statistics
Immuni (Italy)	1 June 2020	+ 4 M (14%)	+ 10 M (16%)	De-centralized	Apple-Google Exposure Notification	Region required	Digital COVID certificate
TousAntiCovid (France)	2 June (launched as StopCOVID)	+ 2.3 M (3%)	+ 32 M (47%)	Centralized	ROBERT (centralized based on legacy BLE)	No information required	Information center QR-code scanning (check-in) Digital COVID certificate
Corona-Warn-App (Germany)	16 June 2020	+ 17.8 M (21%)	+ 32.4 M (39%)	De-centralized	Apple-Google Exposure Notification	No information required	Risk assessment Contact diary QR-code scanning (check-in) Digital COVID certificate
SwissCovid (Switzerland)	25 June 2020	+ 1.5 M (17%)	+ 1.65 M (19%)	De-centralized	DP-3 T and Apple-Google Exposure Notification	No information required	QR-code scanning (check-in)
NHS COVID-19 (UK)	24 September 2020	–	+ 26.8 M (40%)	De-centralized	Apple-Google Exposure Notification	Region required	Symptoms check QR-code scanning (check-in) Test booking

*Percentage of users is calculated with respect to the total inhabitants of the country

Among the first countries to develop and launch a contact tracing app was Singapore with TraceTogether. To date, more than 4.8 million users (i.e., around 90% of the population) have registered on the app or are using the physical tracking tokens equipped with Bluetooth, which were introduced because of the slow uptake of the app at the outset (tracetogether.gov.sg). Based on the same framework, Australia’s COVID Safe app reached a user base of around 7 million, which represents over a quarter of the country’s population, but has been shelved because of performance barriers and technical flaws (SkyNews Australia 2021). Italy, which was one the countries most affected by COVID-19, launched the Immuni app in June, but its adoption rate remains at 16% (Nepori 2021). Switzerland introduced the SwissCovid app in June 2020 and had over 1.6 million users after one year but continues to lag behind its active user goal of 3 million for the app to be effective (FOPH 2021). France launched StopCOVID during the same period but had to release a new version of the app (TousAntiCovid) at the end of October 2020 to overcome adoption barriers. This version has an adoption rate of 47% and owes its success to the added features that include a digital COVID certificate for vaccination. Similarly, Germany’s Corona-Warn-App was launched in June 2020 and has reached over 32 million users (over 39% of the population) after integrating new features (RKI 2021). One of the late arrivals was the UK’s NHS COVID-19 app, which was launched in September 2020 and has an adoption rate of 40% – thanks, in large part, to the additional features integrated into the app (NHS 2021).

### Design of Contact Tracing Apps for COVID-19

The design of national contact tracing apps has been subject to lively debate in most European countries that mostly focused on their privacy implications and the technology design for privacy-preserving apps (Ahmed et al. 2020; Cho et al. 2020; Yasaka et al. 2020). Common tracing mechanisms rely on a smartphone’s absolute location (in the case of location-based tracing) or relative location to other smartphones (in the case of proximity-based tracing) (Legendre et al. 2020). Proximity-based contact tracing relies on Bluetooth Low Energy (BLE) to infer the relative proximity of smartphones (up to 50 m outdoors and 25 m indoors), while location-based contact tracing uses GPS for precise location. Whereas most countries use BLE technology in the design of their contact tracing apps, only a few have adopted a location-tracking mechanism to cross-check paths, as Israeli app Hamagen has done.

The type of architecture adopted for the alerting mechanism in these apps (i.e., centralized versus decentralized) has significant privacy implications (Ahmed et al. 2020). While both approaches require a central server to exchange users’ pseudo IDs, the main difference is the matching of traces with positive user IDs. With the centralized approach, IDs are shared with the central server managed by the public health authorities to match with positive cases and notifications. Doing so allows authorities to have a controlled environment in which to fight the pandemic since the alerting is carried out by the central server in the case of a match. With a decentralized approach, the matching is done on the user’s smartphone with the list of infected IDs. Both approaches communicate anonymously; however, the decentralized approach is regarded as more privacy-preserving since no logging data is exchanged with the server from the user’s smartphone, except in the case of infection (Legendre et al. 2020). While Singapore and Australia follow centralized approaches, the only Western European country to do so is France with the TousAntiCOVID app (originally launched as StopCOVID), which is built based on the ROBust and privacy-presERving proximity Tracing protocol (ROBERT). It is worth noting that apps with a centralized architecture might require preregistration with personal information (e.g., TraceTogether and COVIDSafe) for verification by the central server; however, apps relying on the ROBERT protocol do not require such information (Ahmed et al. 2020).

The core functionality of apps is tracing and alerting users. In addition, these apps can provide features for fighting the pandemic and applying safety measures. For instance, logged data on encounters and information provided on the app can be used to estimate possible infection risk; this is the case for the Corona-Warn-App, which has a risk assessment feature. Other apps provide notifications about safe places and infected zones or contextual services such as check-in services for safe entry (e.g., TraceTogether). With the emergence of vaccines, the digital COVID certificates have been added to Corona-Warn-App and TousAntiCovid. From Table 1, we find evidence that the continuous development and introduction of further features beyond proximity tracking and notifications increased user engagement and adoption since the contact tracing apps’ first launch.

### User Perspective on Contact Tracing Apps

User adoption is a crucial factor for the success of contact tracing apps in curbing the transmission of COVID-19. The public debate led by experts and politicians has mostly focused on privacy concerns as barriers to adoption (Cho et al. 2020). Walrave et al. (2020) highlight the ethical and legal concerns that users have about digital contact tracing and call for a transparent relationship with users and clear processing of their information. Two perspectives prevail in the existing research (Table 2): The first perspective investigates users’ intentions to use contact tracing apps in terms of motivations and barriers (Altmann et al. 2020; Li et al. 2020; Meier et al. 2021; Walrave et al. 2020), and the second applies conjoint analysis to study user preferences for the privacy-aware design of these apps (Degeling et al. 2020; Zhang et al. 2020; Buder et al. 2020; Jonker et al. 2020).

**Table 2:** User perspective on contact tracing apps

Authors	Sample	Method	Focus	Findings
Altmann et al. (2020)	Multiple countries (n = 5995)	Survey	Intention to use	Cybersecurity and privacy concerns, as well as lack of trust in the government, are barriers to adoption
Hassandoust et al. (2021)	US (n = 853)	Survey	Intention to use	Intention to use affected by risk beliefs, perceived individual and societal benefits to public health, privacy concerns, privacy protection initiatives, and technology features Trust in public health authorities affects intention to use
Li et al. (2020)	US (n = 1963)	Survey	Intention to use	Perception of the public health benefits and others’ willingness to adopt has a larger impact on adoption than perceptions of the app’s security and privacy risks
Simko et al. (2020)	Multiple countries (n = 2337)	Survey	Intention to use	Privacy concerns about data sharing, usage, and developer identity limit users’ intention to use Informed consent and transparency can mitigate privacy concerns Technical and legal concepts play an important role in users’ intention to use
Walrave et al. (2020)	Belgium (n = 730)	Survey	Intention to use	Perceived benefits of the app, followed by self-efficacy and perceived barriers, have an impact on adoption rate Cues to action are positively associated with the users’ intention to use
Meier et al. (2021)	Germany (n = 952)	Survey	Intention to use	Perceived benefits of the app are more important than privacy concerns Trust can mitigate privacy concerns and increase perceived benefits
Trang et al. (2020)	Germany (n = 518)	Survey	Intention to use	Citizens with different propensities for acceptance: critics, undecided, advocates In addition to privacy and convenience, multilayered benefit structure is an important factor for mass acceptance
Degeling et al. (2020)	Australia (n = 793/n = 1215) (before/after outbreak onset)	CA-Choice experiment	App design: Privacy features	Relative importance of seven attributes: respect for personal autonomy; privacy; data certainty; data security; infectious disease mortality prevention; infectious disease morbidity prevention; and attribution of (causal) responsibility
Horvath et al. (2020)	UK (n = 1504 and n = 809)	CA-Choice experiment	App design: privacy features	Impact of multiple attributes related to privacy and data security on users’ decision
Zhang et al. (2020)	US (n = 2000)	CA-Choice experiment	App design: privacy features	Attitudes toward six attributes of the hypothetical app: app developer, app name, data storage architecture, expiration conditions, minimum percentage of US smartphone users for effectiveness, and technology use
Buder et al. (2020)	Germany (n = 1472)	CA-Choice experiment	App design: Privacy features and additional benefits	Preferences for the different configurations and importance of attributes with privacy considerations and technology implementation, monitoring, and additional benefits
Wiertz et al. (2020)	UK (n = 2061)	CA-Choice experiment	App design: Privacy features and additional benefits	Preferences for the different configurations and importance of attributes with privacy considerations and technology implementation, monitoring, and additional benefits
Frimpong and Helleringer (2020)	US (n = 394)	CA-Choice experiment	App design: Privacy features and financial incentives	Importance of financial incentives in the decision-making process about app use compared with privacy and accuracy
Jonker et al. (2020)	Netherlands (n = 900)	CA-Choice experiment	App design: Additional benefits and financial incentives	Relative importance of different attributes related to the type of warnings, testing, control over the communication of test results, and financial incentive for app adoption
Behne et al. (2021)	Germany (n = 1993) (n = 53)	Survey (to evaluate existing features) Prototype testing	App design: Additional benefits	Evaluation of enhanced tracing app containing 13 potential front-end (i.e., information on the regional infection situation, education and health literacy, crowd and event notification) and six potential back-end functional requirements (i.e., ongoing modification of risk score calculation, indoor versus outdoor)

When it comes to intentions to use, the results of these studies emphasize the alleviated user concerns about their data privacy and sharing their information with the application owner, whether a private institute or government. A great portion of the discussion has revolved around the application’s architecture, which favors a decentralized approach and type of data shared (e.g., the use of location data) (Li et al. 2020). In addition, Simko et al. (2020) emphasize the role of trust in the government for overcoming privacy concerns associated with contact tracing apps through transparency and clear communication about data management processes within the app. Trang et al. (2020) highlight the need for a clear understanding of the benefit structure to provide insights on the most valuable features that can drive user adoption.

Studies relating to contact tracing app design employ discrete choice experiments in assessing users’ preferences and trade-offs for different implementation options. Alternatively, Behne et al. (2021) employ prototype testing to study enhanced contact tracing apps. The core focus of the conjoint studies is on privacy features related to application architecture and data sharing (Horvath et al. 2020; Zhang et al. 2020). Buder et al. (2020) and Wiertz et al. (2020) employ conjoint analysis to determine the optimal app configuration for an increased adoption rate to above 60% in Germany and the UK, respectively. Their studies demonstrate the role of additional benefits (e.g., priority testing) in improving adoption, in addition to the secure and privacy-aware design. Moreover, Frimpong and Helleringer (2020) and Jonker et al. (2020) find that financial incentives can motivate further downloads of the app and boost its adoption. However, all of these studies limit their scope on app design in terms of privacy-related features.

### Research Gap

Given the slow adoption rates and the criticality of contact tracing apps in this pandemic, but also in the future, there is a pressing need for empirical studies to investigate whether individuals are willing to use these apps and under what circumstances (van Wyl 2020). Prior research strongly focuses on privacy-aware design and the incentives to use these apps (see Table 2) but has missed studying the different facets of actual app designs, including the wider set of services that support users. With the exception of Behne et al. (2021) and O’Connell et al. (2021), we are lacking studies that discuss the learnings from the existing app designs and produce design knowledge that helps improve them to achieve higher user acceptance. From prior IS research and the privacy calculus, we infer that privacy-aware design (covering the risks or costs) should be studied in the context of core and value-added services (offering benefits to users). In addition, we have seen from previous studies that perceived benefits for both self and society, privacy perceptions, and usability aspects can play an important role in the adoption of contact tracing apps (e.g., Trang et al. 2020). In view of the diverging user perceptions, we conclude that analyzing individual and group preferences for different app designs and their privacy trade-offs could provide important insights for prescriptive design (Bélanger and Crossler 2011). From general research on mobile app design, we know that goal-congruent feature additions to core services exert a positive influence on app adoption (Wortmann et al. 2019). Applied to contact tracing apps, the role of value-added services is an area worth exploring to maximize app adoption and, thus, lead to effective countermeasures against COVID-19 and its variants.

## Research Approach and Design

Our study builds on the idea of goal-congruent feature additions as drivers for the adoption of contact tracing apps. It aims to understand individual and group user preferences for different app designs and analyze the impact of value-added services on the adoption of contact tracing apps to improve their design.

We employ CA, which seeks to provide evidence of the factors that most influence the consumer’s choice of a product. Applying the utility concept from economics, CA enables the estimation of a user preference structure based on his evaluation of different product attributes or features. For these reasons, CA is gaining popularity to study information privacy trade-offs in different types of services (Krasnova et al. 2009; Ho et al. 2010) and is a very suitable method to inform IS design through an empirical analysis of user preferences.

In applying CA, we follow the methodological guidelines for IS studies outlined by Naous and Legner (2017, 2021) and use ACBCA, which extends the traditional full-profile CA (Green and Srinivasan 1978). This CA variant combines the advantages of adaptive- and choice-based procedures (Johnson et al. 2003). It is based on a choice experiment where participants have to choose from among a set of profiles (corresponding to different product combinations) after they perform a self-explicated task to assess must-have and unacceptable attribute levels from the evaluation to reduce the choice burden. Based on users’ choices, part-worth utilities and relative importance measures are calculated using the hierarchical Bayes (HB) estimation (Howell 2009).

### Attributes and Levels Selection

The first (and often challenging) step in CA is to identify the attributes that are relevant to forming users’ preferences. In selecting the attributes and levels, we followed a mixed-method approach (Naous and Legner 2017) based on four stages.

In the first stage, we reviewed recent articles that compare the different contact tracing apps (Legendre et al. 2020; Ahmed et al. 2020) and assess the user’s perspective (Trang et al. 2020; Gupta and De Gasperis 2020) in order to identify the attributes describing the core functionalities and privacy-related characteristics. This resulted in 12 attributes corresponding to four dimensions representing the main contact tracing app features: *initiation*, *core functionalities*, *transparency and control*, and *platform characteristics*. In the second phase, we examined nine contact tracing apps (cf. Table 1) to understand the realization options in actual contact tracing apps and identify the attribute levels. Based on this analysis, we decided to add two attributes to our list characterized as *value-added services* that can provide additional benefits and attract more users: diagnosis and contextual services.

In the third phase of the attributes and levels selection, we organized a focus group with five current and potential users of COVID-19 apps to identify important attributes and eliminate the unacceptable option. This phase also allowed us to add one attribute that we did not consider in our initial list. For the app to provide a risk assessment, additional health information might be required for accurate estimations. Therefore, we consider health information registration as an option for the initiation dimension.

Finally, we assess the list of attributes and levels with two privacy experts, who are also familiar with the different contact tracing apps and validated the attributes and the levels. Based on these phases, our final list comprised 10 attributes with corresponding levels (Table 3):

**Table 3:** List of attributes and levels

Dimension	Attribute	Attribute levels
Initiation	Health information registration	No information is required
		Health status (i.e., COVID-19 risk groups information)
Core functionalities	Exposure logging	Contacts (Bluetooth)
		Locations (GPS traces)
		Contacts & locations
	Test results sharing	User can share symptoms or positive test results on app
		User can share positive test results on app only with a validation code obtained from the healthcare provider
		Healthcare provider directly shares test results (positive/ negative) with users
	Exposure notification	Alert only if you had contact with an infected person
		Alert if you had contact with an infected person; includes risk assessment (low, medium, high)
Value-added services	Diagnosis services	No in-app diagnosis
		Simple diagnosis: symptoms tracking with a checklist
		Advanced diagnosis: using sensors to capture symptoms (e.g., breathing, coughing)
	Contextual services	No additional services
		Check-in service with QR code in public places for safe entry (e.g., restaurants, supermarkets)
		Maps that indicate safe areas/infected zones
Transparency and control	Dashboard	Basic dashboard on data logging
		Detailed dashboard on data logging, updates, and sharing
	Data sharing	Restricted to contact tracing (sharing with app provider – in other words, only public health authorities)
		Contact tracing, epidemiological insights, and research (sharing with public health authorities, healthcare providers, and researchers)
		Contact tracing, research, and specific purposes for safety measures (e.g., restaurants, transportation providers, workplace)
Platform Characteristics	Architecture	Centralized
		Decentralized
	Interoperability	Cross-country integration
		No cross-country integration

The *Initiation* dimension specifies attributes related to registering on the app. We excluded attributes for registration options that are present in all apps and focus only on one attribute:

- Health information registration: specifies whether data about health status (e.g., COVID-19 risk groups) is required on the app or not for a more robust data analysis and ideally risk assessment.

*Core Functionalities* comprises three basic features offered by all existing COVID-19 apps:

- *Exposure logging* corresponds to the tracing mechanism employed on the app. It could be proximity tracing with Bluetooth technology, location tracking via GPS traces, or a combination of both.
- *Test results sharing* indicates how the exposure notification is triggered on the app. It could be via user sharing of symptoms or test results. Sharing of test results could be by the user and validated by the healthcare provider, or directly by the healthcare provider (i.e., also includes clearing status in case of a negative test result).
- *Exposure notification* refers to how users get notifications in case of an encounter with an infected person. It could give an alert only in case of exposure; users can also get a risk assessment based on logged data, information on the country region, health status, and other background information.

*Value-added services* comprise features that provide additional benefits to users.

- *Diagnosis services* can be used to check COVID-19 symptoms. They can be either through basic health checklists on possible symptoms or advanced diagnosis with machine learning on mobile sensor data (i.e., heart rate, breathing, coughing strength, etc.) (CORDIS 2020).
- *Contextual services* correspond to additional services related to safety measures; examples are check-in services for safe entry in public places based on customer count or identification of safe places and infected zones through interactive maps.

*Transparency & Control* comprises features for transparent data management on the app.

- *Dashboard* corresponds to transparency about the data usage on the app. It could be a basic dashboard on status and data logs or more detailed with sharing information on data logging, contact traces, and sharing parties.
- *Data sharing purpose* refers to the target of the data sharing and with whom it will be shared. It can be restricted to contact tracing (sharing with app provider – in other words, only public health authorities), involves epidemiological insights and research (sharing with public health authorities, healthcare providers, and researchers), or includes sharing for additional safety measures (e.g., check-in at restaurants, public transports, or workplaces).

*Platform characteristics* relates to the app’s technical design and communication between the app and the remote server.

- *App architecture* corresponds to the alerting mechanism, which can be implemented in a centralized or decentralized approach (Ahmed et al. 2020). In a centralized architecture, users share their IDs with a central server, and matching with positive cases is done on the server. In a decentralized approach, only an infected person is required to share data with the server and all matching with positive cases is done on the user’s smartphone, which periodically receives a list of infected IDs from the server.
- *Interoperability* corresponds to the cross-country integration options. It could be a national app that can only be used in a specific country or allows safe information exchange with other apps to be used when traveling.

### Study Setup

**Fig. 1:**
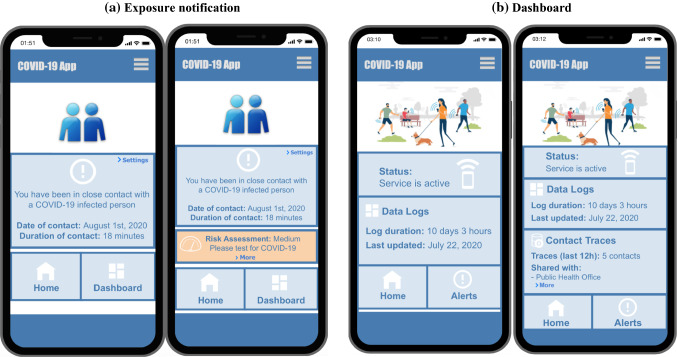
Mobile screenshots for attributes levels

To run our study, we used Sawtooth Software, which is a specialized software with advanced modules for CA survey administration and data analysis. The online survey started with an introduction to contact tracing apps and the conjoint survey sections. We then explained the attributes involved and the different levels (or options) before collecting user choices in the typical ACBCA sections. When possible, we added screenshots of the app to illustrate the differences between levels. This was done for two attributes: exposure notification and dashboard (Fig. 1). Visuals would make it easier for the users to select based on concrete realization instead of verbal descriptions (Naous and Legner 2017).

Participants had to complete the four ACBCA sections in the following order:

1. *Build Your Own (BYO)*. Participants are asked to build the most preferred configurations of the contact tracing app from the list of available attributes and levels. This provides input on individual preferences. Subsequent sections are then adapted to the preferred levels selected by the participants.
2. *Screening*. The survey contained seven screening tasks with three options, where participants assess the possibility of using different app designs. As part of the self-explicated task, this section helps to better understand the user’s non-compensatory behavior. Respondents are asked about must-have and unacceptable features based on their response pattern. To avoid bias in selection, these identified features will not be displayed later.
3. *Choice Task Tournament*. Based on their answers to previous questions, “respondents are evaluating concepts that are close to their preferences in the build your own section specified product, that they consider ‘possibilities’, and that strictly conform to any cut off (must have/unacceptable) rules” (SawtoothSoftware 2014). We present a maximum of 10 choice tasks to respondents with three options. This allows us to estimate the user preferences for the different attributes and levels based on the choice data.
4. *Calibration*. While traditional CBCA includes a “None” option, this is not available in ACBCA. Instead, a “None” threshold can be estimated via the Screening and Calibration section. To calibrate utilities, participants are shown six concepts, including the concept identified in the BYO section, the concept winning the Choice Tournament, as well as four previously shown concepts that were either accepted or rejected. The participant is asked about their likelihood to use these concepts using a five-point scale from “Definitely would not” to “Definitely would

The last phase of the survey included questions on demographics (gender, age) and professional background, as well as questions on general mobile app use and opinion about the COVID-19 app.

### Study Sample

To obtain qualified results, we targeted 300 participants from Germany, the country with the highest number of absolute contact tracing app users (see Table 1), who are users or potential users of the national contact tracing app (Corona-Warn-App). Our choice of this mixture of users and non-users is justified by our research objective, which targets prescriptive design for improved adoption. Our study was conducted in June 2020 shortly after the Corona-Warn-App was launched and users had already become more familiar with its features. Uptake during this initial post-launch period was substantial but flattened in the following months. This allows us to understand opportunities for improvement in the contact tracing app design based on feedback from users. As for non-users, their input is important to build insights on desired app design for potential use.

We selected Prolific.co as a crowdsourcing platform to hire survey participants from an online pool of users. Crowdsourcing platforms, such as Prolific and MTurk, provide a fast, inexpensive, and convenient sampling method and are appropriate for generalizing studies (Jia et al. 2017). They have been widely used in research on security and privacy (Redmiles et al. 2019) and allow a wide reach in CA studies (Pu and Grossklags 2015; Naous and Legner 2019). To guarantee that respondents’ participation was completely anonymous and all data collected would be treated confidentially and not disclosed in its original form, the study setup was examined by the relevant Ethics Committee. Participants were screened based on their smartphone use and knowledge about the COVID-19 app. Survey respondents were compensated £2.50 for their participation, which is a fair amount for a survey between 15 and 20 min on this platform. As quality criteria, we eliminated 17 responses that took less than 7 min for survey completion, which might affect the consistency of the analysis.

Of the total remaining 283 respondents that we included in the final data analysis (Table 4), 55.83% were male and 44.17% were female. Most of the respondents (50.18%) were aged between 26 and 35 years, with 94% younger than 46 years. We assessed their previous privacy experience (based on Xu et al. (2009)) by questioning whether they have frequently heard about the misuse of user information in the media. Based on their responses, the sample can generally be characterized as privacy-aware (82.33%) and reflects the general attitude in Germany, where the population is concerned about misuse of personal information and exposure to social interactions. In terms of mobile app use, our sample is tech-savvy and uses plenty of apps, among them navigation (95.41%), social networking (79.86%), and health and fitness (54.77%). Finally, we note that 62.54% of the respondents think the COVID-19 contact tracing app should be mandatory.

**Table 4:** Sample demographics and background information

Variable	Level	%
Gender	Male	55.83
	Female	44.17
Age	18–25	31.10
	26–35	50.18
	36–45	12.72
	46–55	3.53
	56–65	2.12
	66–75	0.35
Privacy Awareness (based on Xu et al. (2009))	Not informed	17.67
	Well informed	82.33

## Results

### Relative Importance

**Fig. 2:**
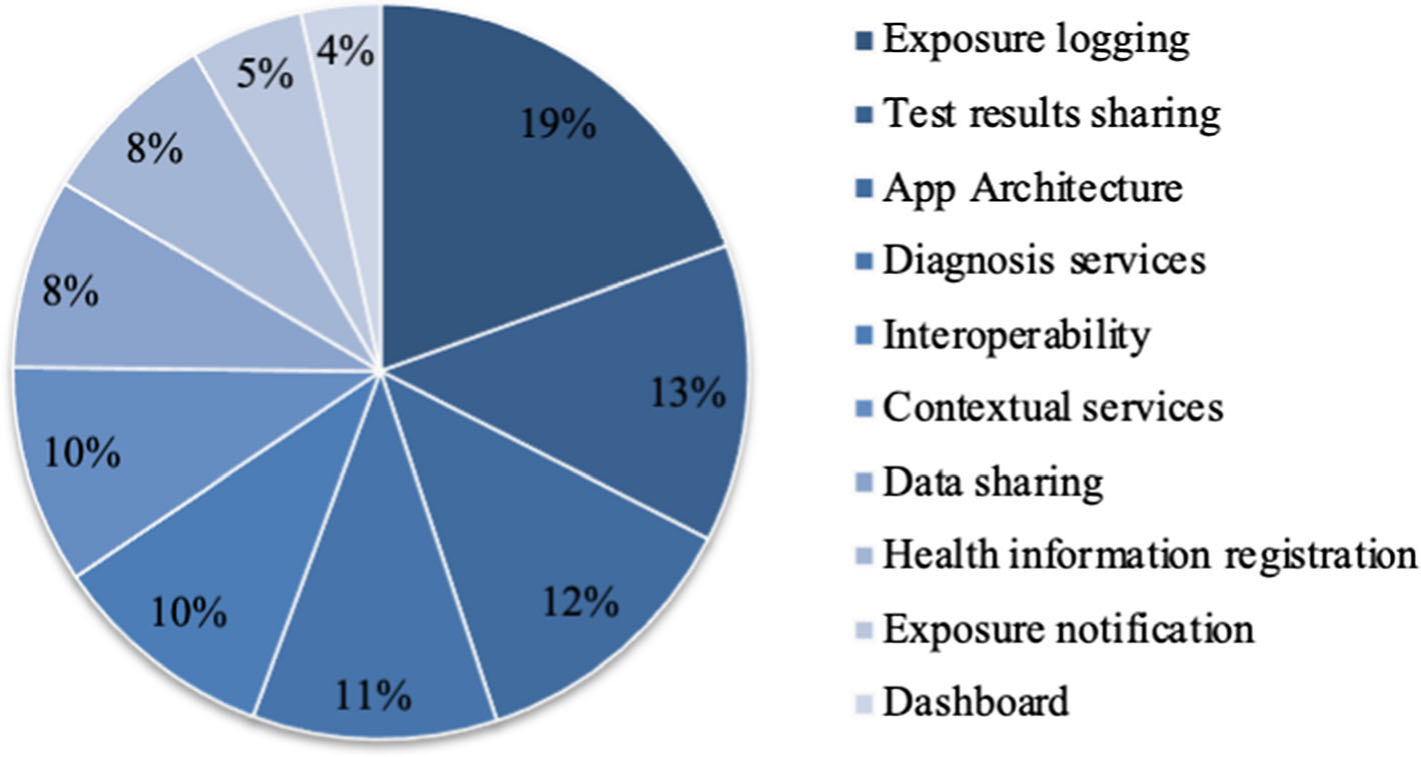
Relative importance of contact tracing app attributes

CA provides relative importance scores based on part-worth utilities for each attribute (Fig. 2). Our results show the contact tracing app’s core services – exposure logging (19%) and test results sharing (13%) – as the two most important attributes. The app architecture (12%) comes next, which reflects the general debate about centralized and decentralized architectures. Diagnosis services had 11%, as a value-added service; interoperability (i.e., cross-country integration) and contextual services had a similar importance of 10%. Data sharing and health information registration follow with an importance score of 8%, despite these two attributes being related to user privacy on the app and the associated risks. Although it is a core service, exposure notification (5%) was less important to users who are not interested in the method or form of notification. Interestingly, transparency on the app was least important with a score of 4%, which contradicts other studies on privacy concerns and transparency in data management (Ahmed et al. 2020).

### Part-worth Utilities and Preferences

Part-worth utilities are normalized HB estimates that provide insight into users’ preferences for the different attributes and levels. Positive utilities correspond to preferred levels, and negative utilities correspond to undesired levels. We assess the “goodness of fit” using percentage certainty (PC) and root likelihood (RLH) (Giessmann and Stanoevska 2012). We obtained a PC mean of 0.486, indicating acceptable results of fit. RLH valued 0.654, which is considered more fit than the chance level given we have three choice tasks.

The part-worth utility distribution (Table 5) allows us to identify attribute levels that are mostly selected by users through the choice options and, thus, correspond to their preference structure and trade-offs regarding the app’s overall design. Interestingly, we observe that users prefer to provide information about their health status on the app, most likely because this information would help generate a more targeted analysis of their COVID-19 status. In terms of exposure logging, contact tracing via Bluetooth (i.e., the most privacy-preserving option) had the highest utility, while GPS tracking had a negative utility and a combination of both has positive utility. For test results sharing, users have positive utilities for trusted and officially validated test results sharing. However, the highest utility was for sharing by the user via a validated code from the healthcare provider. For exposure notification, users appreciate having a risk assessment in addition to the notification.

**Table 5:** User preferences and part-worth utilities (preferred levels are highlighted in bold)

Attribute	Attribute levels	Average utilities	Standard deviation	Distribution for BYO section (%)
Health information registration	No information is required	− 2.86	51.16	43.46
	**Health status**	**2.86**	**51.16**	**56.54**
Exposure logging	**Contacts**	**41.46**	**113.56**	**46.64**
	Location	− 50.00	83.95	15.90
	Contacts and location	8.54	62.07	37.46
Test results sharing	Symptoms or positive test results	− 51.42	58.06	12.37
	**Positive test results with validation code**	**32.69**	**42.74**	**48.06**
	Healthcare provider shares test results	18.72	51.74	39.58
Exposure notification	Contact with an infected person	− 7.01	30.56	40.28
	**With risk assessment**	**7.01**	**30.56**	**59.72**
Diagnosis services	No in-app diagnosis	5.74	53.54	34.63
	**Simple diagnosis**	**25.83**	**31.15**	**47.35**
	Advanced diagnosis	− 31.57	57.32	18.02
Contextual services	No additional services	− 4.52	51.02	37.10
	Check-in service with QR code	− 8.67	52.58	29.33
	**Maps of safe areas/infected zones**	**13.19**	**32.43**	**33.57**
Dashboard	Basic dashboard	− 9.30	18.76	37.81
	**Detailed dashboard**	**9.30**	**18.76**	**62.19**
Data sharing	**Restricted to contact tracing**	**11.12**	**41.96**	**39.93**
	Includes epidemiological insights and research	3.39	26.85	24.38
	Includes specific purposes for safety measures	− 14.51	46.59	35.69
App architecture	Centralized	− 37.37	69.83	37.10
	**Decentralized**	**37.37**	**69.83**	**62.90**
Interoperability	No cross-country integration	− 45.09	44.76	14.13
	**Cross-country integration**	**45.09**	**44.76**	**85.87**

In terms of value-added services, the highest utilities were for simple diagnosis services. Although advanced diagnosis options with mobile sensors can be of great help in detecting patterns and assessing the severity of symptoms, users seem to have concerns about extensive data collection via the app. For contextual services, users prefer the second option, which uses maps to identify infected zones. However, when assessed individually in the BYO section, users stated that they would not prefer an additional contextual service with the app. For transparency and control, higher utilities were recorded for the detailed dashboard and restricted data sharing, which are more privacy-preserving options. For the choice of platform, users have positive utilities for the decentralized approach as a more privacy-preserving approach. Finally, the users prefer cross-country integration. Therefore, our results support the European Union (EU) member states’ effort to establish a technical framework for cross-country contact tracing for travelers and cross-border employees (Lomas 2020).

### User Segmentation

While most research efforts aim for a one-app-fits-all solution, our study reveals various user opinions that need to be taken into account. To gain insights into different user segments for contact tracing apps and varied group preferences, we performed a cluster analysis based on the individual part-worth utilities. Using Sawtooth Software, we employed the Convergent Cluster & Ensemble Analysis module to find optimal groups of users based on their varied preferences. By applying k-means clustering, we were able to derive three clusters of users with varying preferences concerning privacy-preserving features and value-added services (Table 6). The final choice of clusters was based on the highest reproducibility measure, which represents the consistency of the given solution regarding various starting points of the k-means clustering (Orme 2008). While the first two clusters (with a majority of users combined) are concerned about privacy and prefer basic features to guarantee user privacy, the third cluster is unconcerned and would prefer design options that provide an enhanced app experience.

**Table 6:** Identified clusters with preferences based on customer segmentation

	Cluster 1	Cluster 2	Cluster 3
Number of participants	76 (26.85%)	92 (32.51%)	115 (40.64%)
Privacy characterization	Privacy-concerned users	Privacy-concerned users	Unconcerned users
Value-added services	No additional services	Included	Included
*Preferences*			
Health information registration	Not required	Not required	Health status
Exposure logging	Contacts	Contacts	Contacts and location
Test results sharing	Positive test results with validation code	Positive test results with validation code	Healthcare provider shares test results
Exposure notification	Contact with an infected person	With risk assessment	With risk assessment
Diagnosis services	No in-app diagnosis	Simple diagnosis	Advanced diagnosis
Contextual services	No additional services	Maps of safe areas/infected zones	Maps of safe areas/infected zones
Dashboard	Detailed dashboard	Detailed dashboard	Detailed dashboard
Data sharing	Restricted to contact tracing	Restricted to contact tracing	Specific purposes for safety measures
App architecture	Decentralized	Decentralized	Centralized
Interoperability	Cross-country integration	Cross-country integration	Cross-country integration

The first two clusters are similar in terms of their preferences for privacy-preserving features when it comes to the core functionalities, including contact tracing via Bluetooth and sharing only validated test results to avoid false alerts. However, for exposure notifications, the second group prefers having a risk assessment in addition to the notification. The main difference is in the value-added services, where the first segment (26.85%) does not prefer any value-added service, while the second segment (32.51%) prefers at least a simple diagnosis service for tracking COVID-19 symptoms, as well as a contextual service that provides information about infected zones and safe places. For all other features, both segments share the same preferences: They do not prefer to share any health information on the app but do prefer a detailed dashboard and no data sharing with parties other than the public health authorities. They also prefer a decentralized approach but with cross-country integration.

The third cluster, with more than 40% of the participants, prefers enhanced features on all attributes. There are major differences with the previous segments in health information registration, exposure logging, and diagnosis services, where this segment prefers a combination of contact and location tracking, as well as advanced diagnostic services. This segment also has inherent trust in the authorities and would choose all available app features, even if they are privacy-intrusive. This is shown in their choice of test results sharing by the authorities and the centralized approach. In addition, data sharing for this segment can help fight the pandemic in different contexts.

### Variation Analysis

With market simulations enabled by conjoint analysis, we can understand whether adding value-added services with the proposed contact tracing app can result in higher market shares and, therefore, better adoption rates. Specifically, variation analysis allows us to study the effect of changing attributes on market share predictions by comparing utilities for different designs with respect to a reference app. Thus, it provides a market simulation based on reliable quantitative data that can feed the design of the app and identify features that would improve the adoption.

As a reference app, we use the characteristics of the German Corona-Warn-App. We then propose five variations (Table 7) corresponding to the multiple combinations of value-added services within the app. App 1 has a simple diagnosis service for checking symptoms via checklists. App 2 has an advanced diagnosis service based on data processing of sensor data (e.g., heart rate, breathing, coughing, etc.) and applying machine learning algorithms. App 3 has a safe entry check-in service with a QR code that can be used in public spaces to track the number of people inside a place and the positive check-ins. App 4 has a map function with indications of safe places and infected zones within a region. The final app (App 5) combines two value-added services that are selected with the highest utilities: simple diagnosis and map function.

**Table 7:** Scenarios for variation analysis simulatio

Label	Reference	App 1	App 2	App 3	App 4	App 5
Description	Corona-warn-app	Simple diagnosis	Advanced Diagnosis	Check-in service	Maps	Simple diagnosis + maps
Health information registration	No information is required					
Exposure logging	Contacts (via bluetooth)					
Test results sharing	User can share positive test results on app only with a validation code obtained from the healthcare provider					
Exposure notification	Alert if you had contact with an infected person with risk assessment					
Dashboard	Basic dashboard on data logging					
Data sharing	Restricted to contact tracing					
App architecture	Decentralized					
Interoperability	No cross-country integration					
Diagnosis services	No in-app diagnosis	Simple diagnosis: symptoms tracking with checklists	Advanced diagnosis: using sensors to capture symptoms	No in-app diagnosis	No in-app diagnosis	Simple diagnosis: symptoms tracking with checklists
Contextual services	No additional services	No additional services	No additional services	Check-in service with QR code in public places for safe entry	Maps with indication of safe areas/infected zones	Maps with indication of safe areas/infected zones
Market share		41%	28%	35%	40%	43%

Based on the simulation results, we find that all the apps generate market shares. This means their utility is higher than the None threshold,^1^ and people would be willing to adopt such apps. The calculations of the market shares are adjusted to the smartphone user population in Germany to reflect realistic measures of the total population. The difference in market shares compared with the reference app (i.e., Corona-Warn-App) vary in strength. We observe that App 1 (simple diagnosis) and App 4 (Maps) would result in higher market shares within their categories for value-added services, with slightly better results for App 1. Consequently, App 5 with a diagnosis service of symptoms tracking and contextual service of maps also resulted in higher market shares: 43% of users.

## Discussion: The Varying Users’ Preferences for Contact Tracing Apps

The results from our conjoint analysis provide a micro perspective (i.e., that of the user) of users’ preferences for contact tracing apps through an evaluation of feasible design options. In contrast to prior research, our study goes beyond privacy-preserving aspects and the predominant black-box view of contact tracing apps. It provides a system evaluation by using a comprehensive set of features that include core and value-added services, as well as platform characteristics and user control (privacy-preserving features). Our approach helps improve app design and complements existing studies focused on user perception (see Table 2), thanks to the fine-grained assessment that highlights which of the features are required or most valued by users. By delivering data-driven insights that may serve as input for the participatory design of contact tracing apps (Gupta and De Gasperis 2020), our findings contribute to Pillar III of Von Wyl et al. (2020) regarding digital tracing apps.

With regard to individual user preferences, we find that exposure logging and test results sharing are the most important features in contact tracing apps, while exposure notification as the third core service lags far behind. Our findings support the dominant privacy-preserving design of most European contact tracing apps and confirm user preferences for a decentralized approach and contact tracing through proximity rather than location-based tracking via GPS. Despite the ongoing debate about privacy concerns and contact tracing apps, the results show that not all privacy-preserving features are valued by users. Previous research in IS has emphasized the negative impact of users’ privacy concerns on system use (Xu et al. 2009; Krasnova et al. 2010). Acquisti and Grossklags (2004) explain that users’ attitudes can be contradictory and result in a privacy paradox phenomenon. In this phenomenon, according to Barth and de Jong (2017), users are willing to compromise their privacy based on their assessment of the cost–benefit trade-offs. Accordingly, our results provide empirical evidence of privacy trade-offs with regard to contact tracing apps, in which users focus on the benefits associated with the app during a critical worldwide pandemic rather than the privacy risks or costs they entail. An alternative interpretation is that users trust these apps because they implement privacy through design principles and are implemented by authorities who protect the privacy rights of citizens through the EU General Data Protection Regulation (GDPR) (Yang et al. 2020).

Our study also provides interesting insights into the behavior of heterogeneous respondent groups with diverging preferences, represented by the user segments identified. Unlike Trang et al. (2020), we realize that no one app fits all and that different specifications of tracing apps contribute to their mass acceptance. While we observe a trend in favor of privacy-preserving features and basic functionalities (in the first and second segments), the largest user segment deems value-added services more important than privacy-enhancing features. The market simulation and variation analysis illustrate that contact tracing apps achieve higher market shares if value-added services are added beyond the basic app for tracing encounters. This is in line with research by Wortmann et al. (2019), who show that adding goal-congruent features to a core system may result in a higher rate of adoption. For contact tracing apps, the goal-congruent design implies a paradigm shift from a strong focus on privacy-aware design to explore value-added services that complement the app (e.g., through diagnosis and contextual services). Our results are in line with empirical evidence and current developments of the national contact tracing apps (see Table 1), where those countries that have integrated value-added services within the apps show an increase in the adoption rates. For instance, the TousAntiCOVID and the Corona-Warn-App have received several updates over time that included goal-congruent features such as a check-in service and vaccination certificate, which led to a greater user base. Hence, value-added services could become a game-changer in the adoption challenge.

To take privacy preferences into account, a viable implementation option is to integrate auxiliary apps with the COVID-19 app if needed. This is what Singapore did by merging the national TraceTogether app with the SafeEntry app as part of its safety measures to. The move led to an increase in the adoption rate (Lee 2020). A platform design could help avoid that several applications functioning in silos emerge, with none of them achieving critical mass: For example, Switzerland was the first country to promote a decentralized approach that preserves user privacy, but its app design did not have goal-congruent features until 2021, when it added an event check-in option. Individual apps are widely used in Switzerland, such as SocialPass (similar to the Luca app in Germany) for check-ins at restaurants and shops and a COVID Certificate app for vaccination. Applying a strategy similar to the one in Germany by including all these different features into the SwissCOVID app as a single platform can help increase user adoption to ensure broader protection while satisfying heterogeneous user needs.

## Contribution

In conclusion, our study contributes to the design efforts of contact tracing apps in particular and to design research in general. First, we contribute theoretically through emphasizing the role of goal-congruent features in addressing heterogeneous user groups and thereby fostering adoption. Building on the work of Wortmann et al. (2019), we argue that contact tracing app system design can be improved through the integration of value-added services that benefit the self as well as the public. Our results provide an illustrative example of the impact of goal-congruent features on system use and acceptability and help explain the uptake of contact tracing apps in Germany, France and UK. Second, we contribute methodologically by demonstrating the use of CA in exploring preferences with large, heterogeneous user groups and providing insights into trade-offs between core and privacy-preserving features as well as value-added services. Thereby, CA supports the participatory design and can complement existing methods through its different techniques. Specifically, market simulations bring insights into the feature selection and design process of these apps.

From a practical perspective, our results are relevant to the application developers and service providers of contact tracing apps. The preference model resulting from the CA study provides concrete realization options of the contact tracing app to be taken into consideration to gain sufficient critical mass and acceptability among users. Our findings support the development strategies of contact tracing apps, which have extended their services in the meantime with additional benefits (e.g., the German Corona-Warn-App has an integrated check-in function). To support their participatory design (Gupta and De Gasperis 2020), we provide a data-driven approach that allows capturing user preferences and including different stakeholders in the discussion of the most convenient design options. This approach is relevant for contact tracing apps, but can also inform the future design of mobile apps for health and crisis management that rely on sharing sensitive information (Behne et al. 2021).

## Limitations and Outlook on Future Research

Contact tracing apps have a national scope and, thus, may be impacted by both national implementation and contextual factors. As a result, an important limitation of our study is its focus on a sample from Germany, which has specific cultural characteristics and a democratic system, as well as an a priori model of decentralized contact tracing. It would be interesting to look at comparative studies in other countries that have different government regulations and app providers or have introduced centralized proximity or location-based tracking apps to assess the different design options. A cross-country perspective can provide additional insights into user trade-offs, which are governed by contextual and situational circumstances in their country.

Since privacy is a dominant topic in contact tracing apps, we have to acknowledge that our study focused on understanding user preferences for app design, but we did not use a detailed questionnaire to assess our respondents’ privacy awareness or concerns. Nonetheless, the results of the ACBCA clearly showed the intention to use value-adding instead of privacy-preserving features, which reflects the users’ a priori preferences and experiences.

Finally, it is important to note that our study took place while the contact tracing apps were being launched, and our selection of attributes was based on available and suggested options in June 2020. This implies that our CA study did not cover features that were created especially for testing and vaccination. However, the suggested methodological approach could be used to study those additional goal-congruent features in order to further improve the design of contact tracing apps and expand the use of market simulations in application design.

## Footnotes

1 With the ACBCA, a None parameter can be estimated in market simulations to predict whether the respondents would select a proposed option or not. On that basis, if the utility of the product concept proposed is higher than the None utility, it will be chosen.
